# Uveal Melanoma: Current Trends in Diagnosis and Management

**DOI:** 10.4274/tjo.37431

**Published:** 2016-06-06

**Authors:** Berçin Tarlan, Hayyam Kıratlı

**Affiliations:** 1 Private Practice, Ophthalmology, Ankara, Turkey; 2 Hacettepe University Faculty of Medicine, Department of Ophthalmology, Ankara, Turkey

**Keywords:** Eye, neoplasm, uveal melanoma

## Abstract

Uveal melanoma, which is the most common primary intraocular malignancy in adults, arises from melanocytes within the iris, ciliary body and choroid. The diagnosis is based principally on clinical examination of the tumor with biomicroscopy and indirect ophthalmoscopy and confirmed by diagnostic techniques such as ultrasonography, fundus fluorescein angiography and optical coherence tomography. The clinical diagnosis of posterior uveal melanomas can be made when the classical appearance of a pigmented dome-shaped mass is detected on dilated fundus exam. Uveal melanomas classically show low to medium reflectivity on A-scan ultrasonography and on B-scan ultrasonography the tumor appears as a hyperechoic, acoustically hollow intraocular mass. Management of a suspicious pigmented lesion is determined by its risk factors of transforming into a choroidal melanoma, such as documentation of growth, thickness greater than 2 mm, presence of subretinal fluid, symptoms and orange pigment, margin within 3 mm of the optic disc, and absence of halo and drusen. Advances in the diagnosis and local and systemic treatment of uveal melanoma have caused a shift from enucleation to eye-conserving treatment modalities including transpupillary thermotherapy and radiotherapy over the past few decades. Prognosis can be most accurately predicted by genetic profiling of fine needle aspiration biopsy of the tumor before the treatment, and high-risk patients can now be identified for clinical trials that may lead to target-based therapies for metastatic disease and adjuvant therapy which aims to prevent metastatic disease.

## INTRODUCTION

### Epidemiologic Characteristics

Melanoma is a malignant tumor arising from melanocytes and may originate from the skin (91%), the eye and tissues surrounding the eye (5%) or the mucosa (1%).^1^ In 2% of patients, the source cannot be identified.1 Ophthalmic melanomas can arise in the uvea (85%), eyelid/orbita (10%) and conjunctiva (5%).^1,2^ Uveal melanoma is the most common primary intraocular malignancy in adults, and most uveal melanomas originate in the choroid (90%), followed by the ciliary body (7%) and the iris (2%).^3^ The mean age at diagnosis is 60 years and the prevalence is estimated as 4.9 per million men and 3.7 per million women.^4,5,6,7^

Although the treatment approach has shifted from enucleation toward more eye-conserving therapies over the last 20 years, the 5-year survival rate has remained stable (about 81.6%). In addition to an increasing preference for therapeutic modalities that conserve the eye, there is also a growing trend toward early treatment of tumors classified as small melanomas instead of monitoring.^4,7^

### Predisposing Factors

Both host and environmental factors influence the development of uveal melanoma.

### Host Factors

Significant risk factors for uveal melanoma include white race, fair skin and light iris color.^8^

### Melanocytic Lesions Associated with Melanoma

**Choroidal nevus:** Choroidal nevi are found in 3% of individuals over 30 years old and studies indicate that annual rates of malignant transformation can vary from 1 in 4,300 to 1 in 8,845.^9,10^

**Ocular/Oculodermal melanocytosis:** Ocular or oculodermal melanocytosis is a condition characterized by hyperpigmentation of the episclera, uvea and skin, and is more common in black, Hispanic and Asian populations. Its prevalence in whites is 0.04%, and 1 in 400 cases develops uveal melanoma.^11^

**Cutaneous nevus:** Case-control studies have shown that cutaneous nevi may be a risk factor for uveal melanoma and that patients with dysplastic nevus syndrome have a higher incidence of uveal melanoma.^12,13 ^ This highlights the need for dermatologic evaluation in uveal melanoma patients.

**Familial uveal melanoma:** Recently, an autosomal dominant hereditary cancer syndrome has been described in some patients with germline BAP1 mutation. Patients with this mutation have higher incidences of uveal melanoma, cutaneous melanoma, atypical Spitz tumors, mesothelioma, meningioma, adenocarcinoma of the lung and many other cancer types.^14,15^

### Environmental Factors

**Sunlight:** In contrast to cutaneous melanomas, ultraviolet light has not been shown to play a role in the development of uveal melanoma, except as a result of occupational exposure, as with arc welders.^13,16^

**Diet, smoking and alcohol consumption:** To date there are no studies showing that dietary factors, cigarette use or alcohol consumption have an effect on the incidence of uveal melanoma.

### Diagnostic Methods in Uveal Melanoma

The diagnosis of uveal melanoma is based primarily on clinical examination by biomicroscopy and indirect ophthalmoscopy. In contrast to the basic principles of oncology, histological or cytologic evaluation is not routinely used in the diagnosis of intraocular neoplastic lesions. Ancillary tests including color fundus photography, ultrasonography (USG), fundus fluorescein angiography (FFA), indocyanine green angiography (ICGA), optical coherence tomography (OCT), fundus autofluorescence (FAF) and ultrasound biomicroscopy (UBM) can be used in order to confirm diagnosis. Fine-needle aspiration biopsy (FNAB) of the tumor can be performed when the clinical diagnosis is unclear, and the diagnosis can be clarified by the evaluation of an experienced ocular pathologist. There are currently no clear indications regarding the surveillance and initiation of treatment for small choroidal melanocytic lesions and with the recent understanding that cytogenic findings are among the main prognostic factors for uveal melanoma patients in terms of metastatic disease, biopsies are increasingly performed following diagnosis.^17,18^

Studies of delays in the diagnosis of uveal melanoma found that 28-37% of these lesions were not detected in the first examination. Therefore, it is imperative that patients exhibiting any symptoms suggestive of posterior segment pathology, such as photopsia, metamorphopsia or vision loss, undergo a dilated fundus examination.^19,20,21,22^

The classic appearance of posterior uveal melanoma (ciliary body and choroidal melanoma) is a brown, dome-shaped mass, but it may also appear as mushroom-shaped (20%) or diffuse type (5%). While 55% of the tumors are pigmented, 15% are nonpigmented and 30% include both pigmented and nonpigmented areas.^3,23^

Iris melanomas occur most frequently in the inferior quadrant (45%), are pigmented in 82% of cases and show one of three growth patterns: nodular, diffuse or tapioca.^24^

Unlike iris melanomas, which are clearly visible on clinical examination, ciliary body melanomas may be hidden behind the iris and be difficult to detect, especially when small. Similarly, choroidal melanomas may escape notice without a careful dilated fundoscopic examination. Documenting the size and location of the tumor by color fundus photography is crucial during follow-up in order to evaluate signs of malignant transformation, primarily documented growth.

Posterior uveal melanomas are generally graded based on tumor thickness in research and clinical settings. In this grading system, small tumors are those up to 3 mm thick with a base diameter not exceeding 16 mm, medium tumors are 3.1-8 mm thick with a base diameter not exceeding 16 mm, and large tumors are thicker than 8 mm and have a base diameter larger than 16 mm.^23,25^ It has been established that the risk of metastasis increases 5% with each 1 mm increase in tumor thickness as measured by USG.^23^ Cancer staging classifies the extent of disease based on clinical, pathologic and genetic factors. In the American Joint Committee on Cancer (AJCC) tumor node metastasis staging system, tumor size is evaluated and defined in the T category (1-4), lymph node involvement in the N category (NX, N0, N1) and presence of distant metastases in the M category (MX, M0, M1a, M1b, M1c) (Table 1). For posterior uveal melanoma, T is classified based on tumor basal width and thickness (T1, T2, T3, T4) and then divided into subgroups reflecting ciliary body involvement and extrascleral extension of the tumor (a, b, c, d, e). Studies have showed that this classification system can predict prognosis, and 5-year survival rate of iris melanoma patients was estimated to be 100% for patients with T1 tumors, 90.4% for patients with T2 tumors, 63.6% for patients with T2a tumors and 50% for patients with iris melanomas classified as T3, T3a or T4.26 The metastasis rate of posterior uveal melanoma at 10 years was found as 15% for T1 tumors, 25% for T2 tumors, 49% for T3 tumors and 63% for T4 tumors.^27^

Small melanomas may present as flat or dome-shaped tumors. With time the melanoma ruptures Bruch’s membrane and forms its pathognomonic mushroom shape, which can be easily visualized on USG. Vitreous hemorrhage may also be evident if the tumor has infiltrated the retina after Bruch’s membrane rupture.^28^

USG is the auxiliary method most often used clinically in the diagnosis of uveal melanoma. The tumor typically shows low to medium internal reflectivity on A-mode USG and appears as an acoustically hollow mushroom- or dome-shaped choroidal mass on B-mode USG. In A-mode, the low to medium internal reflectivity of the tumor decreases toward the sclera. This allows discrimination from hemangioma, which typically shows high reflectivity in this mode. In B-mode, tumors appear as a hyper-echoic mass with lower reflectivity than the surrounding choroid, thus giving an acoustically hollow appearance. Choroidal excavation may also be evident, which is more common in large tumors, and orbital shadowing may be observed as well.^29,30^ USG is also useful in the evaluation of extraocular extension; areas of hyporeflectivity compared to normal orbital tissue are considered orbital extension of the tumor.^28^

UBM is useful for the evaluation of tumors which originate from the ciliary body. This technique allows the visualization and evaluation of hyporeflective plaques on the tumor surface, tumor-specific vasculature, internal reflectivity and, if present, extraocular extension.31 Anterior segment OCT is a newer technique used in the imaging of iris and ciliary body melanoma, but it does not yield the same results as USG due to the lack of penetration into deeper tissues.32 In the absence of these auxiliary imaging methods, transillumination, gonioscopy, and oblique biomicroscopy, which allows the visualization of the tumor while the patient looks in the direction of the lesion, can assist visualization of ciliary body melanomas.^28^

Uveal melanomas feature intrinsic tumor circulation as well as choroidal circulation. The observation of this double circulation pattern or leakage from tumoral vasculature is occasionally necessary in order to confirm the diagnosis. FFA, which can visualize these features, is an important technique during the differential diagnosis from other lesions. FFA is also used in the detection and follow-up of complications arising after brachytherapy such as radiation retinopathy and radiation maculopathy.

**Figure 1:**
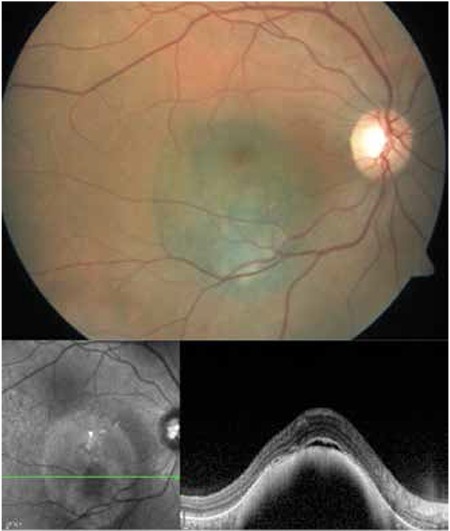
Color fundus photograph and spectral domain-optical coherence tomography image of choroidal melanoma in the posterior pole of the right eye before treatment

OCT can be utilized in ocular oncology as an auxiliary test in diagnosis, treatment planning and evaluating treatment response. Spectral domain OCT (SD-OCT) allows the detailed evaluation of changes in the retina and retinal pigment epithelium overlying lesions in choroidal melanoma. Choroidal melanomas are usually easily distinguished from choroidal nevi based on size, but this distinction may be difficult with lesions less than 3 mm thick. In such cases, OCT can facilitate the detection of features like subretinal fluid, which is considered one of the high-risk features predicting transformation into melanoma.^33,34,35^ With newly developed imaging methods such as enhanced depth imaging it is now possible to examine deeper tissues like the choroid and sclera. With this technique, choroidal nevi appear as dome-shaped or flat lesions causing deep choroidal shadowing depending on the pigmentation of the tumor. The retinal pigment epithelium overlying the mass may be atrophied or absent, with choriocapillaris compression and photoreceptor loss in this area. Although choroidal melanomas may exhibit all of these characteristics, studies have demonstrated that shaggy photoreceptors and subretinal fluid are indicative of choroidal melanoma in the differential between nevus and melanoma (Figure 1).^33,34,35^

On FAF imaging, pigmented tumors exhibit moderate hypoautofluorescence, whereas nonpigmented (amelanotic) tumors show moderate hyperautofluorescence. In both types of tumors, the areas of hyperautofluorescence can be seen due to the presence of orange pigment, drusen and subretinal fluid overlying the tumor. Presence of orange pigment can be confirmed by comparing these hyperautofluorescent areas to the suspicious lesions seen in fundoscopic examination. This method may also reveal hypoautofluorescent retinal pigment epithelium defects, such as hyperplasia, atrophy and fibrous metaplasia, or hyperautofluorescent drusen, both of which indicate chronic stable nevus.^36^

Computed tomography (CT) and magnetic resonance imaging (MRI) can be utilized when tumor visualization by clinical examination presents a challenge, as in patients with media opacities like cataract, vitreous hemorrhage or retinal detachment. Patients with unilateral cataract in particular should be carefully evaluated for uveal melanoma, keeping in mind that ciliary body melanoma may cause unilateral or asymmetric cataract via pressure exerted on the lens.^30^ In patients with unilateral hypermature cataract, it should be kept in mind that dense cataract can resemble ciliary body melanoma on oblique imaging of the lens. Pseudomelanoma due to hypermature cataract can be identified using USG by the presence of an echodense cortex forming anterior and posterior borders, lack of contiguity with the uvea, and ring melanoma-like visibility in all four quadrants.^37^ USG is the first choice when a mass cannot be visually evaluated due to media opacity; CT and MRI can be utilized if a differential diagnosis is still not possible after USG. These imaging methods also have an important role in the evaluation of extraocular extension. On CT it appears as a hyperdense mass with mild/moderate contrast and distinct margins. On MRI, the tumor characteristically returns a hyperintense signal on T1-weighted images and hypointense signal on T2-weighted images. However, this can also be observed in the subacute phase of a limited hemorrhage, causing it to mimic an intraocular mass. Circumscribed choroidal hemangioma is also included in the differential diagnosis, especially of amelanotic melanomas. Like choroidal melanoma, hemangiomas have a hyperintense signal in T1-weighted MRI, but on T2-weighted images they are isointense to the vitreous. These imaging methods are not strictly necessary in the diagnosis stage, but are a requirement in the planning stage of proton beam therapy or stereotactic radiotherapy (SRT).^28^

Intraocular tumors can be biopsied by several methods. Anterior segment tumors can be evaluated by aqueous humour sampling, incisional or excisional biopsy. FNAB (transscleral, transvitreal or transcameral), vitrectomy biopsy, incisional or excisional biopsy (endoresection or transscleral resection) can be done in order to evaluate posterior segment intraocular tumors.^38^

Studies on tumor doubling time of choroidal melanoma indicate that micrometastases occur several years before diagnosis.^39,40^ Unlike cutaneous melanoma, uveal melanoma spreads via the blood and not via the lymphatic system, unless there is invasion of the conjunctiva by the tumor. Extraocular extension occurs hematogenously by penetration into the vortex veins and emissary channels.^41^ Small melanomas are usually preexisting small nevi monitored for growth, and 6-8% of diagnosed uveal melanomas originate from nevi.^21,28^ Considering that most patients never undergo an ophthalmologic examination prior to their uveal melanoma diagnosis, the actual rate of nevus to melanoma transformation is certainly higher. Singh et al.^42^ found malignant transformation of choroidal nevus at a rate of 1 in 8,845 patients. Therefore, clinicians should monitor existing choroidal nevi in consideration of established risk factors predictive of tumor growth. High-risk factors predictive of growth of suspicious pigmented choroidal lesions into melanoma include presence of symptoms, tumor thickness greater than 2 millimeters, presence of subretinal fluid and orange pigment, tumor margin within 3 mm of the optic disc, ultrasonographic hollowness, and absence of halo.^43,44,45^

Posterior uveal melanoma can be confused with many lesions of the retina, retinal pigment epithelium and choroid. According to studies and case reports, the most commonly confused lesions are, in order of frequency, choroidal nevus, peripheral exudative hemorrhagic chorioretinopathy, congenital retinal pigment epithelium hypertrophy, hemorrhagic detachment retinal or retinal pigment epithelium, circumscribed choroidal hemangioma and age-related macular degeneration (AMD).^46^ AMD, extramacular disciform lesions, spontaneous subretinal hemorrhage, polypoidal choroidal vasculopathy and various lesions such as arterial macroaneurysm that present with hemorrhage may simulate choroidal melanoma. During clinical examination, it should be kept in mind that patients with AMD may exhibit macular changes in the fellow eye, and tests such as FFA and ICGA may aid differential diagnosis by revealing intrinsic tumor circulation of choroidal melanoma.^46^

Clinical prognostic factors in uveal melanoma include older age, male gender, increased tumor size, tumor location, diffuse growth pattern of the tumor, presence of extraocular extension, and progression of tumor stage according to AJCC classification. Histopathological risk factors related to prognosis include epithelioid cell type, increased mitotic activity, increased tumor infiltrating macrophages and lymphocytes, expression of human leukocyte antigen and insulin-like growth factor receptor-1, and types of extravascular matrix patterns.^3,23,47,48^ In 1931, Callender^49^ first classified uveal melanoma by histologic cell type as spindle type A, spindle type B, fascicular, epithelioid, and mixed.^50^ That classification system was later modified by McLean et al.50 and uveal melanoma is currently histologically classified as one of three histologic subtypes: spindle cell, epithelioid cell, and mixed cell types. It is generally accepted that epithelioid cell melanoma is associated with the worst prognosis and spindle cell melanoma with the best.^49,50,51^

In their study evaluating metastatic death in 847 uveal melanoma patients, Coupland et al.^52^ reported tumor base width, epithelioid cells, mitotic rate and extraocular extension as the clinical and histopathologic factors with prognosticative value. Eskelin et al.^53^ studied tumor doubling time and found that clinically detectable metastases appear at most 5 years after treatment, but claimed that metastases may be observed up to 25 later because micrometastases begin forming years before treatment.

Studies utilizing immunohistochemical techniques to better understand tumor and microenvironmental characteristics have demonstrated that chemokine receptor CCR7 is strongly expressed in uveal melanoma cells and is associated with systemic metastasis.^54,55^

### Differential Diagnosis

Uveal melanoma is divided into iris, ciliary body and choroidal melanoma, and certain lesions should be considered in the differential diagnosis of these subtypes. Possible diagnoses of a suspicious iris lesion other than iris melanoma include iris nevus, iris pigment epithelial cyst, iris stromal cyst, metastatic tumor of the iris, melanocytoma, iris atrophy and Cogan-Reese syndrome. In addition to melanoma, the differential diagnosis of ciliary body tumors should include staphyloma, medulloepithelioma and leiomyoma. The majority of uveal melanomas are choroidal melanoma, which can be simulated by a variety of lesions. Among these are choroidal tumors, especially choroidal nevus, metastatic tumors, choroidal hemangioma, and osteoma; hemorrhagic conditions like AMD and hemorrhagic choroidal detachment; retinal tumors such as congenital retinal pigment epithelium hypertrophy and retinal pigment epithelium adenocarcinoma; and inflammatory lesions like posterior scleritis.

### Systemic Metastasis in Uveal Melanoma

In a study of 8,033 uveal melanoma patients, Shields et al.^24^ found systemic metastasis rates of 8%, 15% and 25% at 3, 5 and 10 years, respectively. In relation to tumor size, metastases were seen in 12% of small melanoma, 26% of medium melanoma and 49% of large melanoma at 10 years follow-up. In the Collaborative Ocular Melanoma Study (COMS), the 5- and 10-year metastasis rates in choroidal melanoma patients were 25% and 34%, respectively, independently of tumor size.^56^ Examination of the link between age and metastasis rates revealed systemic metastasis at 10 years in 10% of uveal melanoma patients 11-20 years old, 21% in patients 41-50 years old, and 30% in patients 71-80 years old. These results support the opinion that the systemic metastasis rate increases with advancing age in uveal melanoma.^3^ Systemic metastases are most commonly observed in the liver (93%), lungs (24%) and bones (16%).56 After the formation of metastasis, survival time depends on the location of the metastasis. Patients with liver metastases survive for an average of 4-6 months, with a 1-year survival rate of 10-15%. Reported survival time for patients with other metastases is 19-28 months.^57,58^

### Prognosis of Uveal Melanoma

Although tumor size is currently considered the primary factor affecting prognosis, the importance of histopathologic factors and, in recent years, genetic factors has also gained recognition.

### Genetic Indicators

#### Cytogenetic and Molecular Cytogenetic

Despite advances in diagnosis and treatment, uveal melanoma continues to be a life-threatening malignancy, and systemic metastasis is seen in approximately half of patients during long-term follow-up.^23^ Studies in the last 20 years have concentrated on mutations and their molecular basis which may have a role in the pathogenesis of uveal melanoma and formation of systemic metastasis. Genetic alterations in uveal melanoma are investigated using methods such as karyotyping, single nucleotide polymorphism, fluorescent in situ hybridization, microsatellite analysis and comparative genomic hybridization at the deoxyribonucleic acid (DNA) level, and with gene expression profiling (GEP) at the ribonucleic acid (RNA) level.^59,60,61,62,63^

The first reported chromosomal alteration to be associated with poor prognosis in uveal melanoma was chromosome 3 monosomy.^64,65,66^ In subsequent studies, this chromosomal abnormality was found to be associated with epithelioid cell type, presence of microvascular loops, tumor base diameter, ciliary body involvement and cancer-related death caused by metastasis.^59,60^ Chromosome 3 loss is usually accompanied by gain in chromosome 8 (8q).^64,66,67^ Gain in chromosome 8q, especially together with monosomy 3, has been shown to be associated with high metastasis risk.^68,69^ In a study including 452 choroidal melanoma patients, Damato et al.^68^ used multiplex ligation-dependent probe amplification to compare patients with disomy 3, monosomy 3 and both monosomy 3 and 8q gain, and reported 10-year melanoma-related mortality rates of 0%, 55% and 71% in the three groups, respectively.

Other genetic abnormalities that have been described include loss of chromosome 1p and gains in chromosome 6q and chromosome 8p.^70^ Patients without chromosome 3 abnormalities usually exhibit gain of chromosome 6p, and this finding is associated with good prognosis.^71,72^ A study evaluating histopathologic characteristics by karyotype analysis demonstrated that the addition of chromosome 3 loss and chromosome 8q gain to extraocular extension (extrascleral) significantly decreases metastasis-free survival.^73^

### Molecular Genetic

In RNA-based GEP studies evaluating the messenger RNA (mRNA) expression of many genes, uveal melanoma could be divided into two groups: patients with low metastasis risk (class 1) and patients with high metastasis risk (class 2).^62,63,74^ This classification was proposed by Onken et al.,^63^ who determined 8-year survival rates as 95% in class 1 and 31% in class 2. They later transferred their GEP technique to a polymerase chain reaction-based assay analyzing mRNA expression of 12 discriminator genes and 2 control genes in order to create a more clinically feasible standardized test.^63,75^ Trolet et al.^76^ performed array-based comparative genomic hybridization on 86 patients with uveal melanoma and 66 patients with liver metastasis; one of their important findings was that liver metastases were found in 14% of patients determined as class 1.

A variety of steps, such as tumor suppression, G-protein mediated signal transduction, adhesion molecule expression and retinoic acid response, have been investigated in order to elucidate the molecular pathways involved in uveal melanoma. Although less than 1% of all uveal melanoma cases are familial, studies have demonstrated that these patients carry many mutations, primarily germline mutation of BAP1, and have suggested that these mutations may be responsible for transmission in familes.^14,15^

Activation of the mitogen activity protein kinase (MAPK) pathway plays a role in the development of many cancers, especially melanocytic neoplasms.^77^ This pathway can be activated by various mechanisms, and activation via RAS and B-RAF gene mutations is common in cutaneous melanoma. Although MAPK pathway activation has also been reported in uveal melanoma, B-RAF or RAS mutations are rare. However, some studies have attributed this to limitations in the techniques used and claim to have detected B-RAF mutations in uveal melanoma.^77,78,79^

G protein-coupled receptors function with G proteins, which have various alpha (a) subunits. One of these subunits, called Gq or Gqq/11, is encoded by the GNAQ and GNA11 genes. Daniels et al.^80^ found that 91% of patients with large melanoma had GNAQ (47%) or GNA11 (44%) mutations, and Van Raamsdonk et al.^81^ detected GNAQ mutations in patients with Nevus of Ota and uveal melanoma. Somatic mutations in GNAQ or GNA11 activate mitogen-activated kinase (MEK), phosphoinositide 3-kinase/protein kinase B, protein kinase C and yes-associated protein related pathways, which has led to an increasing number of clinical trials targeting these pathways for patients with metastatic uveal melanoma. These oncogenic mutations are seen in the early stages of tumorigenesis, as in benign uveal nevi, and are not associated with molecular class (class 1 or 2) or with metastasis.^81,82,83,84,85^ Clinical studies have demonstrated improved survival of metastatic uveal melanoma patients with selumetinib, a MEK pathway inhibitor, compared to temozolomide.^86^

The BRCA-1 associated protein-1 (BAP1) tumor suppressor gene, located on chromosome 3p21, codes the ubiquitin carboxy-terminal hydrolase enzyme which is among the enzymes responsible for tumor-suppressing activity in cancer cells, and regulates the activity of some proteins through deubiquitination. For example, histone H2A regulates the expression of certain genes; deubiquitination of the BAP1 region is a critical step in tumor-suppressing function. Somatic mutation in this gene is observed in many cancers including breast, lung and mesothelioma and germline mutations have been found in familial uveal melanoma and mesothelioma cases.86,87 It has been shown that BAP1 mutation appears in the late stages of tumorigenesis, causes changes in phenotype, and is associated with metastatic behavior and class 2 genetic structure in 84% of patients.^88,89^ Mutation of one allele is usually accompanied by loss of the other entire copy of chromosome 3.

In contrast to the previously described mutations, splicing factor 3b subunit 1 (SF3B1) gene mutations have been found at a rate of 19% in uveal melanomas and are associated with good prognosis.90 Mutations in this gene also occur later in tumor progression and are relatively specific to class 1 tumors. However, it was shown that patients with disomy 3 tumors and SF3B1 mutations have increased risk of metastatic disease at a longer follow-up time (Koopmans AE, Prognostic implications of acquired genetic changes in uveal melanoma. Unpublished data, Erasmus University, Rotterdam, Netherlands).

Another mutation associated with good prognosis is the eukaryotic translation initiation factor 1A (EIF1AX) gene mutation, which has been reported in 24% of uveal melanomas.^91^

### Therapeutic Approaches in Uveal Melanoma

Treatment for uveal melanoma is initiated at the time of diagnosis and is not limited to the intraocular tumor alone. Management of uveal melanoma also includes the assessment of prognostic factors currently used in clinical practice and, when necessary, the planning of adjuvant therapies targeting systemic disease; post-treatment monitoring and control of recurrence and possible treatment-related ocular side effects; visual function assessment and visual rehabilitation using appropriate treatment options; routine systemic evaluation for metastasis risk; and psychiatric evaluation. Neglecting any one of these steps may lead to treatment failure ending in mortality, despite successful treatment of the tumor.

The currently accepted and clinically applied understanding of tumor management begins with a proper evaluation of prognostic factors, after which one or more therapies are chosen in consideration of these factors to both control the tumor and minimize impact to healthy tissues. Decisions regarding which treatment options are appropriate and applicable are made based on tumor size, location and extension and take into consideration the patient’s preferences and expectations.

Overall prognosis should be evaluated as a combination of prognosis determined by host genetic factors and ocular and systemic prognosis according to treatment options administered.

The two main treatment options for uveal melanoma patients without systemic metastasis are eye-conserving therapies and enucleation. Studies have demonstrated that despite developments in treatment methods and the increasing tendency toward eye-sparing therapies over the last 30 years, survival rates have remained constant. This indicates that successful local treatment of the eye does not affect survival. Therefore, identifying patients at risk of metastasis and referring them to adjuvant therapies in addition to local treatment is crucial.

### Primary Tumor Treatment

**Should every tumor be treated?**

To date, the traditional approach when faced with a small, pigmented choroidal tumor has been monitoring the lesion until findings on color fundus photography indicate growth. However, as it is impossible to know whether a tumor will become metastatic before it reaches a size requiring treatment, delaying treatment may result in metastatic spread. On the other hand, considering that 30-40% of small melanomas are in close proximity to the optic disc and macula, treating all suspicious choroidal tumors would result in unnecessary ocular morbidity and vision loss.^92^

Therefore, small tumors should be evaluated in the context of factors identified in the literature as indicating malignant transformation, and decisions should be made after fully informing patients of the benefits and risks involved in treatment.^43,44,45,87^ Instead of observation, the current opinion favors initiating treatment when risk factors are present; observation at regular intervals is still considered appropriate in a small minority of cases.

In the COMS, tumors with thickness less than 3 mm and basal diameter up to 16 mm tumors were classified as small melanomas, and 204 choroidal melanoma cases were evaluated in the small melanoma arm of the study. During follow-up, 21% of these patients required treatment at 2 years, 33% at 5 years, and 38% at 7 years. Melanoma-related mortality was 1% at 5 years and 3.7% at 8 years.^93,94^ Enucleation and local treatment of the tumor by brachytherapy are controversial because of their impact on visual acuity as well as the low rate of melanoma-related mortality in the long term with small melanomas.

### Eye-Conserving Therapies Photocoagulation

#### Therapies Photocoagulation

Photocoagulation was frequently used in the past to treat small choroidal melanoma, first with xenon arc and later with argon laser photocoagulation. Despite superior tumor control with xenon arc photocoagulation, the argon laser results in fewer complications.95 Today, small tumors less than 3 mm thick and located more than 3 mm from the fovea are treated with transpupillary thermotherapy (TTT).^96,97,98^

### Transpupillary Thermotherapy

**Figure 2:**
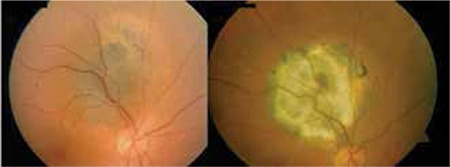
Color fundus photographs of choroidal melanoma before and after treatment with transpupillary thermotherapy

TTT is a diode laser-based method used to treat small and medium-sized melanomas (tumor thickness less than 4 mm).96,97,98,99 In their meta-analysis, Singh et al.^100^ found an average recurrence rate after primary TTT in small melanoma patients of 17% (8-56%) and reported that 7% of these recurrences involved extrascleral extension. In brief, patients undergoing TTT alone for uveal melanoma should be selected carefully, keeping in mind that although visual prognosis is good, there remains the long-term possibility of recurrence with high metastatic risk (Figure 2).

### Radiotherapy

Radiotherapy is currently the most common treatment for uveal melanoma, especially posterior uveal melanoma. In clinical application, radiotherapy can be administered in the form of radioactive plaque, external beam radiotherapy or SRT.

**Figure 3:**
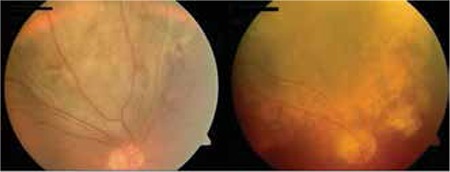
Color fundus photographs of choroidal melanoma before and after treatment with radioactive plaque brachytherapy (Iodine-125)

Brachytherapy is the direct irradiation of a tumor via the application of a radioactive source (radioisotope) to the tumor surface or interior.^101^ There are two types of radioactive sources used in brachytherapy, gamma- or X-ray emitting isotopes and beta-particle-emitting isotopes. Of the isotopes most commonly used in ophthalmic radioactive, Cobalt-60, Palladium-103 and Iodine-125 are gamma sources and Ruthenium-106 (Ru-106) is a beta-particle source. Ru-106 plaques have been found effective for small and medium tumors (basal diameter up to 16 mm and thickness up to 8 mm) when applied alone or in combination with TTT (Figure 3).^102^

The medium tumor arm of the COMS included tumors 2.5-10 mm thick with a basal diameter less than 16 mm and compared patients treated by I-125 plaque brachytherapy versus enucleation.^103^ There was no significant difference between the two groups in 10-year mortality. Melanoma-related mortality rates at 5, 10 and 12 years were 10%, 18% and 21% in the brachytherapy group, versus 11%, 17% and 17% in the enucleation group.

**Figure 4:**
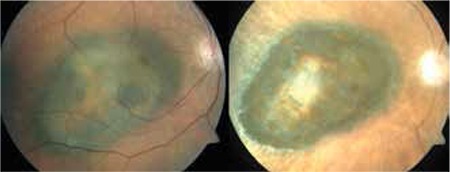
Color fundus photographs of choroidal melanoma before and after treatment with radioactive plaque brachytherapy (Ruthenium-106)

Finger et al.^104^ treated 400 uveal melanoma patients with Palladium-103 and found a metastasis rate of 6% after 51 months of follow-up. They also estimated the 5- and 10-year survival rates as 7.3% and 13.4%. Another study evaluating patients treated with Ru-106 plaques observed local tumor recurrence in 3.9% and reported estimated metastasis rates at 5 and 10 years of 30.9% and 68.2%.^102^ Studies using Ru-106 plaques have shown that this isotope carries an increased risk of local recurrence with tumors with thickness over 5 mm.^106^ For tumor thicknesses between 5 and 8 mm (with basal diameter not exceeding 16 mm), Ru-106 plaque brachytherapy can be utilized but should be supported by the application of TTT to the tumor apex (Figure 4).^102,105,106^

### External Beam Therapy

External beam therapy is the irradiation of a tumor with charged particles such as proton and helium ion beams or with stereotactic methods.^107^ This modality can be used to treat tumors up to 14 mm thick with a basal diameter up to 28 mm.

### Proton Beam Therapy

Unlike brachytherapy and fractionated SRT, proton beam therapy delivers a homogenous dose of radiation to the entire tumor and due to the Bragg effect the radiation dissipates quickly beyond the edge of the target.^108,109^ This allows the delivery of a high dose of radiation to the tumor while preserving adjacent normal tissue; however, tissues in the path of the beam as it enters the body and targets the tumor also receive a high dose of radiation. In theory, all uveal melanomas could be treated by proton beam therapy but for large melanomas the visual prognosis and eye conservation rates remain low.^110^ The first choice of treatment for large tumors located in the superotemporal quadrant should be radioactive plaque radiotherapy in order to spare the lacrimal glands. In a series of 2,413 melanoma patients treated with proton beam therapy, Desjardins et al.^111^ reported 5- and 10-year metastasis rates of 18.5% and 26.6%, respectively. Local recurrence was observed in 4% of the patients at 5 years and 10% at 10 years, with most occurring in the first 3 years after treatment. After a follow-up period of about 8 years, complications noted were loss of eyelashes in 12%, retinal detachment in 8.5%, glaucoma in 23.4%, dry eye in 6%, cataract requiring surgery in 15%, optic neuropathy in 18% and maculopathy in 37% of the patients. TTT was used as an adjuvant therapy when the tumor was close to the macula or to decrease the likelihood of neovascular glaucoma, and recurrence was not observed in these patients.

### Stereotactic Radiotherapy

SRT is the irradiation of a tumor with a photon beam. In SRT the radiation is delivered as a single dose, while in fractionated SRT the total dose is delivered as smaller equal doses. An advantage of treating with a stereotactic photon beam is that no surgical procedure is required to determine the tumor’s location, the tumor borders are determined by MRI and CT.^112,113^ Although proton beam therapy (charged particle therapy) is theoretically superior in terms of sparing healthy tissue from the effects of radiation, stereotactic radiosurgery might be more advantageous in certain cases since it does not require preoperative surgical marking and is more cost-effective.^114,115^

The devices used in stereotactic photon beam irradiation are the Gamma Knife, linear accelerator, and the CyberKnife. Ocular immobilization is required for both stereotactic radiosurgery and SRT techniques. This can be achieved with retrobulbar anesthesia or vacuum-assisted immobilization frame for the Gamma Knife and the cameras used to monitor eye movements for the linear accelerator.^113^

### Gamma Knife

First applied in the treatment of brain tumors, the Gamma Knife has since been used to treat uveal melanomas with successful results.^116,117^ However, it is not a preferred treatment modality due to high reported rates of radiation retinopathy and neovascular glaucoma (8.6-64%).^118,119,120,121^ The main problem with this technique is ocular fixation. Zehetmayer et al.^122^ used the Gamma Knife to treat 62 uveal melanoma patients unsuitable for Ru-106 plaque brachytherapy and reported higher morbidity with tumors larger than 8 mm and a dose of 10 Gy/fraction was a high risk factor for radiation-induced inflammation.

### CyberKnife

**Figure 5:**
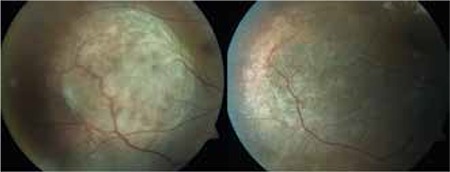
Color fundus photographs of choroidal melanoma before and after treatment with CyberKnife (stereotactic radiosurgery)

CyberKnife radiosurgery was also first used in brain surgery and is currently utilized to treat uveal melanoma. Zorlu et al.^123^ used CyberKnife to treat 5 patients who were not eligible for plaque radiotherapy or local resection and reported that 3 patients showed reduction in tumor size at 8-month follow-up. In the same center, 163 uveal melanoma patients were treated with CyberKnife (stereotactic radiosurgery/fractionated SRT). During the follow-up period of mean 24.2 months (range, 2-79 months), local control was achieved in 74% of patients and progression was observed in 17.2% (Yazıcı et al., submitted for publication) (Figure 5).

**Linear accelerator:** The linear accelerator is used to treat uveal melanoma by stereotactic hypofractionated radiotherapy. The advantages of this approach are less radiation exposure to the healthy tissues adjacent to the tumor and avoidance of long-term effects. Noninvasive fixation systems designed for use with linear accelerators have increased patient comfort and compliance with treatment.^113^

### Complications of Radiotherapy

#### Radiation Retinopathy

**Figure 6:**
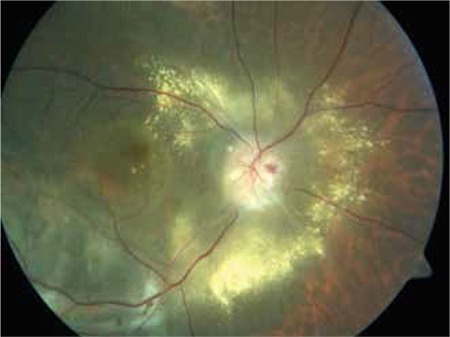
Color fundus photograph of a choroidal melanoma patient with radiation retinopathy and maculopathy

Radiation retinopathy is a chronic, progressive vasculopathy of the retinal capillaries resulting from radiotherapy-induced damage to the vascular endothelium.^124^ This damage causes capillary dilation, increased vascular permeability, thrombosis, retinal exudate and hemorrhage, eventually leading to full thickness retinal atrophy (Figure 6). Capillary non-perfusion is evident on FFA, the gold standard in the diagnosis of radiation retinopathy.^125^ Radiation retinopathy is observed in 42% of patients 5 years after brachytherapy, and usually occurs in the first 2 years after treatment.^126^ The first sign may be a decrease in visual acuity due to subclinical macular edema, and in fact the detection of macular edema on OCT is an indicator that radiation maculopathy may develop an average of 5 months later.^127,128^ Retinopathy is dependent on the total radiotherapy dose received and the area of the retina irradiated. It is generally accepted that retinopathy develops rarely with radiation doses under 35 Gy and it occurs in about half of patients receiving 65 Gy or more.^129^ Ischemic retinopathy can often progress to proliferative retinopathy, which may be observed at an average of 2.5 years after plaque radiotherapy, and vitreous hemorrhage may occur in 15.1% of patients at 5 years after treatment.^130,131^ Guyer et al.^132^ reported the incidence of radiation maculopathy after proton beam radiotherapy as 90%.

Treatment options include panretinal or focal laser photocoagulation, photodynamic therapy, intravitreal or periocular triamcinolone injection, oral pentoxifylline, hyperbaric oxygen, intravitreal anti-vascular endothelial growth factor (anti-VEGF) and intravitreal silicone application prior to brachytherapy.^133,134,135^ Missotten et al.^136^ showed that VEGF-A levels are higher in the aqueous humour of both treated and untreated uveal melanoma patients. Taking into account that VEGF levels are elevated in uveal melanoma and further elevated in radiation retinopathy, Shah et al.^137^ administered intravitreal bevacizumab (Avastin) injections for 2 years to 292 of 418 patients treated with plaque radiotherapy and observed the other 126 patients without further treatment. They observed lower rates of OCT-evident macular edema and radiation retinopathy during follow-up in the patients treated with bevacizumab.

### Radiation-Induced Optic Neuropathy

Radiation-induced optic neuropathy typically causes sudden, painless, unilateral vision loss starting as early as 3 months or up to 8 years after radiation exposure.^138,139^ Since the pathogenesis of the optic nerve damage is not fully understood, radiation-induced optic neuropathy is considered to be radionecrosis of the optic nerve and chiasm. This presents clinically as optic disc edema with lipid accumulation and hemorrhages; later, optic atrophy with ghost vessels are observed. Systemic and intravitreal corticosteroids, hyperbaric oxygen therapy, anticoagulant therapy, and intravitreal anti-VEGF therapy have been used in treatment.^139,140,141,142^ Finger and Chin^139^ treated 14 choroidal melanoma patients who developed radiation-induced optic neuropathy after radioactive plaque therapy with intravitreal anti-VEGF injections (at least 2 injections at 6-8 week intervals) and reported regression of optic disc edema and improvement of papillary hemorrhage.

### Surgery

#### Enucleation, Exenteration, Local Resection

Although enucleation was the most common treatment choice in the past, it is currently reserved for cases with the worst visual prognosis, such as patients with large uveal melanoma (tumoral thickness greater than 8 mm), with choroidal melanoma surrounding the optic nerve, or presenting with severe hemorrhage, retinal detachment or vitreous hemorrhage. There is no consensus on the maximum tumor thickness that can be treated by radiotherapy. If the COMS is accepted as a guide, the plaque applied should exceed the tumor margins by 2 mm. In practice, episcleral application of a plaque larger than 25 mm is not possible, limiting the use of plaque radiotherapy to tumors with a maximum basal diameter of 21 mm. Only medium melanomas were treated with plaque radiotherapy in the COMS; for large melanomas, enucleation alone or in combination with preoperative external beam therapy were applied. There was no significant difference in the 10-year survival rate between the group that received preoperative radiotherapy and the group that did not.^143^ Studies in which large tumors (basal diameter over 16 mm and thickness greater than 8 mm) were treated with plaque radiotherapy have reported poor visual prognosis even when the eye is spared.^144,145^

In terms of survival, many studies have demonstrated no significant difference in mortality between eye-conserving and surgical treatment approaches. Comparison of the COMS medium uveal melanoma patients treated with plaque brachytherapy and those that underwent enucleation revealed no significant difference in long-term survival.^145,146^ Therefore, in recent years eye-conserving treatments have gained favor over enucleation.

Local resection is an alternative treatment choice for choroidal melanoma patients which spares the eye and, more importantly, allows a detailed histopathologic and cytogenetic analysis. The procedure is more preferred in cases of iris and ciliary body melanoma. Iridectomy is currently the first choice for iris tumors, and is indicated in tumors covering up to a third of the iris but not extending to the angle.^147^ However, proton beam therapy may be preferable to iridectomy in order to avoid the resulting surgical coloboma.^148^ Iridocyclectomy is indicated for tumors with angle or ciliary body involvement.^147^ Choroidectomy is currently only performed by a small number of surgeons due to the technical challenges. Tumors can be surgically removed via a transretinal (endoresection) or transscleral (exoresection) route. Plaque radiotherapy is recommended as an adjuvant to exoresection to prevent tumor recurrence, though the preventative application of plaque radiotherapy before endoresection is still controversial.^149,150^ Major complications such as retinal detachment and vitreous hemorrhage have been reported with both techniques. Tumor location within one disc diameter of the optic disc has been reported as the most important risk factor for severe vision loss following local resection.^151^

Exoresection is recommended in cases of toxic tumor syndrome, a condition in which the irradiated tumor becomes ischemic and exudative, resulting in macular edema, exudation, serous retinal detachment, uveitis, rubeosis iridis and neovascular glaucoma.^151^

### Systemic Evaluation

There is no definitive guideline or even a consensus regarding screening tests for systemic metastasis in uveal melanoma. In particular, clinical examinations for the presence of subcutaneous nodules and organomegaly are imperative. Liver function tests include gamma-glutamyl-transpeptidase, lactate dehydrogenase, alkaline phosphate, aminotransferases and bilirubin levels. Because abnormal liver function test results have lower sensitivity and specificity compared to radiographic investigation, these tests should only be used as a complement to radiography.^152^ Metastases undetectable by liver function tests may be evident on USG.^153,154^ Contrast MRI is the most sensitive method for liver imaging; CT is highly sensitive but its ability to discriminate from benign lesions is weak.^155,156^

Just as in deciding the course of treatment, it is important to determine the best clinical approach in order to provide individualized patient care and risk stratification for a patient diagnosed with uveal melanoma. The clinical and histopathologic prognostic factors can be used to separate patients into general risk categories, but they are not accurate enough to be used for individualized patient care. Furthermore, chromosomal alterations including chromosome 1p loss, chromosome 3 loss, chromosome 8q gain and chromosome 6p gain might be used for clinical prognostification, but it was shown that they need to be considered together with clinical and histologic risk factors.^68^

As mentioned above, GEP of uveal melanoma allows the accurate discrimination of primary tumors at low metastatic risk (class 1 signature) and high metastatic risk (class 2 signature). The gene expression test was developed for routine clinical use and has been validated in a prospective, multi-center study which reported that the GEP test had prognostic accuracy that was superior to clinicopathologic staging and monosomy 3.^157,158,159^ Field and Harbour^158^ recommended annual liver imaging for patients in class 1A (low-risk) based on GEP analysis, evaluation every 6 months alternating between liver imaging and liver function tests for class 1B (intermediate-risk) patients, and evaluation every 3 months alternating between liver imaging and liver function tests for class 2 (high-risk) patients.

### Adjuvant Therapies

Despite advances in the diagnosis and treatment of uveal melanoma, the general mortality remains high due to metastatic disease. It is therefore extremely important to identify patients at high risk for metastasis, and many studies have investigated this topic in recent years. On the other hand, studies of tumor doubling rate have provided evidence that uveal melanomas metastasize before diagnosis. Detection of circulating tumor cells in the bloodstream at time of diagnosis further support this.^39,160,161,162^ Metastatic uveal melanoma is resistant to treatment, and there is no evidence that current treatment is able to extend survival. The efficacy of systemic treatment could be improved with adjuvant therapies that target micrometastases instead of macrometastases.

Adjuvant therapies consist of radiotherapy and systemic therapy, which currently target clinically identified macrometastases. Systemic treatment options include chemotherapy, immunotherapy, hormone therapy, biologic therapy and targeted therapy. Unlike other tumors, there are still few studies regarding uveal melanoma. Non-randomized studies conducted so far have utilized dacarbazine, bacilli Calmette-Guerin and systemic interferon, but have not reported promising results.^163,164,165^ Uveal melanoma patients were treated with systemic interferon alpha-2a subcutaneously 3 times a week for 2 years, but there was no significant difference in mortality.^163^ Studies of sunitinib, valproic acid, dacarbazine, systemic interferon alpha-2b, vaccination with dendritic cells and ipilimumab as adjuvant therapies are ongoing, and results have yet to be reported.^166^ Fotemustine, an alkali cytotoxic chemotherapeutic agent, has been used to treat patients with liver metastasis as both intravenous chemotherapy and as intra-arterial hepatic chemotherapy. Intra-arterial hepatic administration resulted in better tumor response than systemic administration but did not increase survival in the long term.^167^ The MAPK pathway, activated by GNAQ mutations plays an important role in the pathogenesis of uveal melanoma. Although the MEK-inhibitor selumetinib did not improve survival in cutaneous melanoma, when administered to uveal melanoma patients with GNAQ mutation it extended progression-free survival.^168,169^ The main limitation of MAPK inhibitors is that the drug is effective for an average of 6-10 months and it is believed this leads to more aggressive recurrences.

Studies on preventing metastasis and extending survival in high-risk uveal melanoma patients are currently in progress. Ipilimumab is being evaluated as a systemic adjuvant therapy following treatment of the primary tumor in patients identified as class 2 by RNA analysis, exhibiting monosomy 3 on DNA analysis, or having a tumor over 8 mm in thickness.^170^ Patient enrollment has concluded for a clinical trial evaluating treatment response to dacarbazine and recombinant interferon alpha-2b in patients who exhibit monosomy 3 or 8q gain without any metastasis, but results have not been published yet.^171^ Similarly, the c-Ros oncogene inhibitor crizotinib is being administered to patients with genetic class 2 tumors in a phase 2 clinical trial, but the patient recruitment is ongoing and no results have been published.^172^ A clinical trial of sunitinib and valproic acid in genetically high metastasis risk uveal melanoma patients is in progress and currently recruiting participants.^173^ The inhibition of the hypoxia-inducible factor pathway by arylsulfonamides is another area of continuing research.^174^

In recent years, proteomics and secretomics studies aiming to enable the early detection of metastasis have gained importance. Osteopontin, S100, MIA (melanoma inhibitory activity), VEGF and TPS (tissue polypeptide specific antigen) are among the proteomic markers shown to be elevated in the serum of metastatic uveal melanoma patients. Elevated expression of S100, MCAM, NKI-C3, E-cadherin, c-Met and MIA has been demonstrated in the tissue of metastatic melanomas.^175^

In conclusion, despite advances in the diagnosis of uveal melanoma and treatment for localized disease, up to half of patients will develop fatal metastatic disease. With progress in understanding the molecular landscape of the tumor and the development of treatments targeting the pathways involving GNAQ/GNA11, BAP1, EIF1AX, SF3B1 mutations and epigenetic mechanisms, in the near future it may be possible to prevent the progression of micrometastases.

## Ethics

Peer-review: Externally and internally peer-reviewed.

## Figures and Tables

**Table 1:**
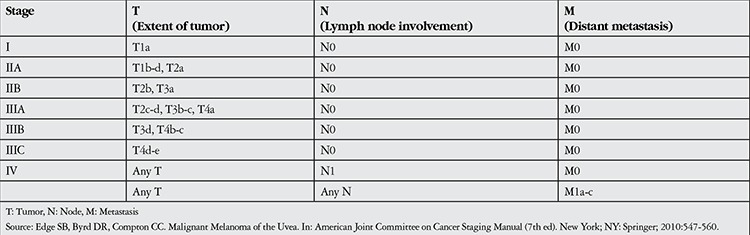
American Joint Committee on Cancer staging of uveal melanoma based on extent of tumor-node- metastasis
